# Oscillometrically Measured Aortic Pulse Wave Velocity Reveals Asymptomatic Carotid Atherosclerosis in a Middle-Aged, Apparently Healthy Population

**DOI:** 10.1155/2020/8571062

**Published:** 2020-01-16

**Authors:** Renáta Marietta Böcskei, Béla Benczúr, Veronika Müller, András Bikov, Andrea Székely, Thomas Kahan, Zsófia Lenkey, Róbert Husznai, Attila Cziráki, Miklós Illyés

**Affiliations:** ^1^Heart Institute, Medical School, University of Pécs, Pécs, Hungary; ^2^Department of Pulmonology, Semmelweis University, Budapest, Hungary; ^3^1st Department of Internal Medicine, Balassa Janos County Hospital, Szekszárd, Hungary; ^4^Department of Anesthesiology and Intensive Care of Semmelweis University, Budapest, Hungary; ^5^Department of Clinical Sciences, Danderyd Hospital, Division of Cardiovascular Medicine, Karolinska Institutet, Stockholm, Sweden

## Abstract

**Background:**

Asymptomatic atherosclerosis is a common entity even at young age. Studies have suggested a strong relationship between increased arterial stiffness and asymptomatic carotid atherosclerosis (ACA) in general population, particularly in those with high cardiovascular risk, but no data exist from a younger population free from recognized cardiovascular disease. *Hypothesis*. We hypothesized there is an association between ACA and aortic pulse wave velocity (PWVao) in middle-aged, apparently healthy, normotensive population to reveal increased cardiovascular risk.

**Methods:**

We examined the relationship between ACA and PWVao in 236 apparently healthy, asymptomatic, normotensive, middle-aged subjects (age 47 ± 8 years; 52% women). PWVao was measured with the oscillometric method (Arteriograph). ACA was assessed by carotid artery ultrasonography.

**Results:**

ACA was present in 51 subjects. Subjects with ACA were older (*p* < 0.009), more likely to be smokers (*p* < 0.009), more likely to be smokers (*p* < 0.009), more likely to be smokers (*p* < 0.009), more likely to be smokers (*p* < 0.009), more likely to be smokers (*p* < 0.009), more likely to be smokers (*p* < 0.009), more likely to be smokers (*p* < 0.009), more likely to be smokers (

**Conclusions:**

PWVao measured by the Arteriograph proved to be an independent marker of ACA. Our study may reveal high CV risk, detected as increased PWVao, which according to our study is related in a very high probability to asymptomatic carotid atherosclerosis in apparently healthy, young, and middle-aged subjects.

## 1. Introduction

Identifying asymptomatic atherosclerosis in apparently healthy, middle-aged patients has high clinical impact to detect increased cardiovascular risk [1, 2]. The Systematic Coronary Risk Estimation (SCORE) has its limitations regarding the prediction of future cardiovascular (CV) events as more than 80% of the events occur in patients of low- and middle-income countries, where the prognostic power of SCORE has not investigated and validated [3]. Furthermore, young and middle-aged population is more prone to nonfatal CV events than fatal cardiac diseases so that the SCORE can hardly be used as an accurate tool for predicting overall CV risk as it determines only CV mortality. Furthermore, the SCORE cannot be used under 40 years of age although the prevalence of aortic atherosclerosis was found to be 62.5% and 61.5% in men and women aged 30–34 years [4].

The gold standard diagnostic tool to reveal an asymptomatic carotid atherosclerosis (ACA) is the ultrasound examination of the extracranial part of both carotid arteries. Many studies emphasize the added value of measuring and characterizing carotid plaques beside intima-media thickness (IMT) when predicting CV risk [5, 6]. Despite the hardly questionable evidence of an early onset atherosclerosis gained by the PDAY Study [4], moreover the known low performance of SCORE to predict CV events in this population, the 2016 European Society of Cardiology Guidelines on cardiovascular disease prevention in clinical practice concludes that “routine screening with imaging modalities to predict future CV events is generally not recommended in clinical practice and should only be used as a risk modifier in individuals with calculated CV risks based on the major conventional risk factors around the decisional thresholds” [7]. Consequently, an easy-to-perform, low-cost, validated method that can identify apparently healthy middle-aged patients with high cardiovascular risk by detecting asymptomatic atherosclerosis would have great clinical importance.

Aortic pulse wave velocity (PWVao) is a widely accepted marker of high CV risk, and it has an additive value in risk assessment beyond traditional risk factors [8, 9]. Former studies, demonstrating a strong relationship between PWVao and ACA [10–12], have made PWVao a promising method to reveal high risk subjects with ACA [13]. However, the generally accepted and most frequently used applanation tonometric and piezoelectric methods to measure aortic PWV may not really be suitable for the clinical practice because of their time-consuming, sophisticated manner and they also require trained personnel.

According to our knowledge, no publication is available which investigated the relationship between ACA detected by ultrasound and regional (aortic arch to bifurcation) aortic stiffness, measured as aortic PWV in middle-aged, apparently healthy, normotensive subjects. However, it has also been proven that early detection of a target organ damage such as ACA representing high CV risk can lead to earlier and more effective therapeutic strategies [14, 15].

Therefore, the aim of our study was to examine the association between ACA and PWVao with a clinically easily implementable, simple and fast oscillometric method in middle-aged, apparently healthy, normotensive population to reveal high CV risk.

## 2. Methods

### 2.1. Study Population

**Figure 1:**
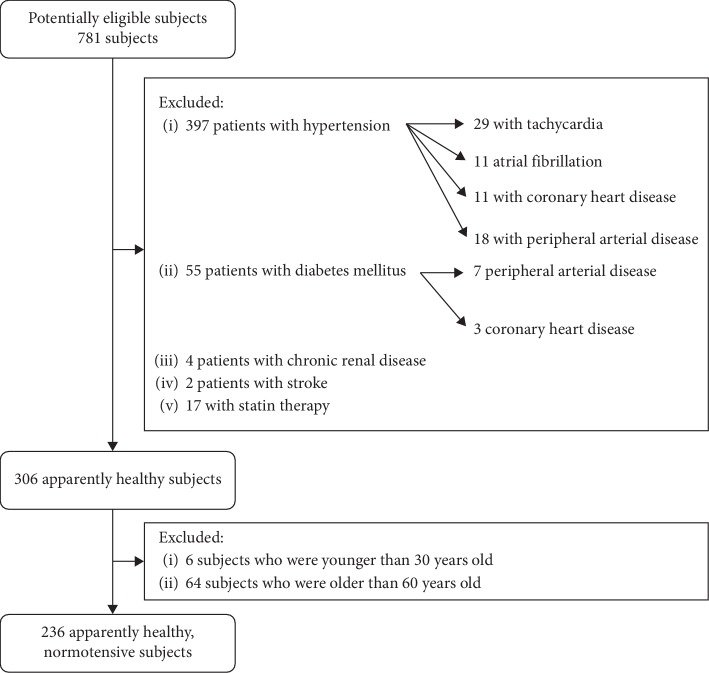
Flow chart of the number and selection of individuals in the study population.

In this observational cross-sectional study, we measured a relatively big cohort, 781 subjects (aged 57 ± 12), who attended our daycare center of Heart Institute, Medical School, University of Pécs, Hungary, by their own initiative for a cardiovascular checkup.  Figure 1 displays the enrollment of participants into the study.

In this study, apparently healthy, young, and middle-aged subjects were included, provided they were without any complaints or case history for coronary heart disease or stroke. Exclusion criteria of this cross-sectional study were coronary heart disease, hypertension, atrial fibrillation, tachycardia (heart rate above 90beats/min), stroke, peripheral arterial disease, chronic kidney disease, diabetes mellitus, and statin use. Those patients who were previously diagnosed with coronary heart disease (e.g., positive case history, or who had experienced for angina pectoris or who had positive coronary angiography/positive coronary CT scan) were excluded. Hypertension was defined as systolic blood pressure ≥140 mmHg, diastolic blood pressure ≥90 mmHg, or current use of antihypertensive medication. Diabetes mellitus was defined as a current use of oral antidiabetic drugs, insulin, or a self-reported diagnosis. Chronic kidney disease was excluded by anamnestic data and according to laboratory parameters (GFR lower than 60 ml/min/1.73 m^2^). Current smoking status was self-reported. Thus, 236 (age 47 ± 8 years; 52% women) apparently healthy, normotensive subjects remained for statistical analyses. Baseline characteristics, including patient history, physical examination, and serum total cholesterol values were obtained by standard procedures. The study was been approved by the local Institutional Ethics Committee of the University of Pécs, Pécs, Hungary (PTE KK RIKEB—5111/2013), and all participants gave their oral and written informed consent.

### 2.2. Carotid Artery Ultrasound Examination

All carotid artery ultrasound examinations were performed by one single examiner using a HP Sonos 2000 device with a 7.5 MHz linear probe (Hewlett Packard Ltd., Andover, Massachusetts, USA) on the same day and within 1 hour of the measurement of arterial stiffness. The whole extracranial carotid artery system was examined bilaterally, and we followed the American Society of Echocardiography (ASE) guidelines regarding standard screening methods of carotid plaques [5]. Near and far walls of all arterial segments (common carotid artery, carotid bifurcation, and the origins (first 2 cm) of the internal carotid arteries and external carotid arteries) were scanned longitudinally and transversally to assess the presence of asymptomatic carotid artery atherosclerosis. Asymptomatic carotid atherosclerosis (ACA) was defined according to the European Mannheim consensus [16]. The presence of a plaque in the carotid artery was defined as a focal structure encroaching into the arterial lumen of at least 0.5 mm or 50% of the surrounding IMT value or demonstrates a thickness >1.5 mm as measured from the media-adventitia interface to the intima lumen interface.

### 2.3. Aortic Stiffness

The simultaneous measurements of PWVao and blood pressure were performed in the supine position after 10 minutes of rest by an invasively validated oscillometric, occlusive, noninvasive technique (Arteriograph, TensioMed Ltd., Budapest, Hungary). This method allows very simple, fast, and user-independent measurement of aortic PWV using only a single upper arm cuff. The variability and reproducibility of the Arteriograph measured aortic PWV reported to be superior compared to applanation tonometric and piezoelectric methods [17]. Details of the method and its invasive validation have been published previously [18]. In brief, the device first measures the actual brachial blood pressure oscillometrically with a clinically validated algorithm [19] and then inflates the cuff to suprasystolic pressure, completely occluding the brachial artery. During this condition, pure pressure signals can be collected by the cuff with pronounced and easily detectable direct and reflected systolic wave peaks. The time difference between the early and late systolic peaks is equal to the time of the aortic pulse wave traveling down to the aortic bifurcation and back towards the heart. From this, the aortic root-bifurcation transit time can be calculated, and by measuring the straight distance between the suprasternal notch and pubic bone (an acceptable estimate of the aortic length), the PWVao can be calculated.

As an estimate for the measurement errors for the repeat measurements of PWVao with Arteriograph, the variance within one session was 0.18 m^2^/s [2] and between two sessions was 1.18 m^2^/s^2^ [17].

### 2.4. Statistical Analyses

Data are reported as mean values ± SD. Group means of continuous variables were compared with independent samples Student's *t*–test, whereas groups of categorical variables were analyzed with the *χ*^2^ test. Stepwise logistic regression was used to define predictor variables for the binary outcome of the presence of ACA. Receiver operating characteristics (ROC) analysis was performed to assess threshold values for PWVao relative to ACA. A probability (*p*) < 0.05 was considered to be significant. Data were analyzed using SPSS 16.0 statistical package (SPSS Inc., Chicago, Illinois, USA).

The sample size was estimated to detect differences in PWVao between patients with and without ACA with an effect size of 0.38 and power of 0.80. The effect size was based on the distribution and the variability of PWVao data [17].

## 3. Results

236 asymptomatic normotensive subjects (age 47 ± 8 years; 52% women) comprised the study group. The participants' characteristics are presented in Table 1. ACA was present in 51 subjects (22%).

There were no differences between subjects with and without ACA in gender, BMI, total cholesterol, or HR. However, age, smoking, SBP, and PWVao were higher in the ACA group compared to ACA negative subjects (50 ± 8 vs. 47 ± 8 years, *p*=0.009; 29% vs. 8.3%, *p* < 0.001; 128 ± 9 mmHg vs. 125 ± 10 mmHg, *p*=0.048; 9 ± 2 m/s vs. 8 ± 1 m/s, *p* < 0.001, respectively). A stepwise logistic regression including age, gender, BMI, smoking habit, SBP and DBP, HR, and PWVao was used to reveal parameters independently related to ACA. PWVao, smoking habits, and SBP and DBP were independently associated with ACA (Table 2). Age was not an independent marker of ACA. Based on the calculated odds ratio (1.88) and the SD (1.6 m/s) of PWVao, 1 SD increase of PWVao almost duplicated (98%) the risk of ACA, supposing that other parameters remained unchanged.

**Figure 2:**
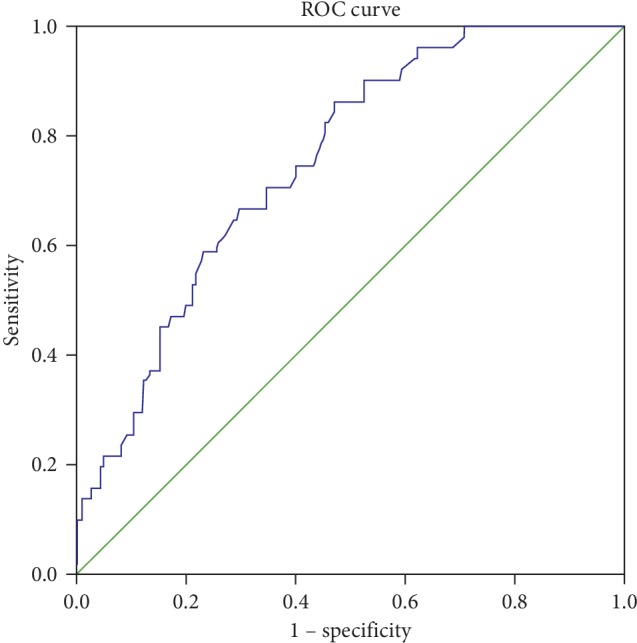
Receiver operating characteristic curve determining asymptomatic carotid atherosclerosis by measuring aortic pulse wave analysis.

A receiver operating characteristic (ROC) curve analysis was performed to determine the optimal threshold value of PWVao for detecting ACA. This value proved to be 8.3 m/s (Figure 2).

The area under the curve was 0.751, and the optimal PWVao threshold proved to be 8.3 m/s, with a sensitivity of 0.71 and a specificity of 0.65 (see Table 3 for details).

Using this cutoff value of PWVao, the sensitivity, specificity, positive and negative predictive values, relative risk, and odds ratio for ACA were 0.71, 0.65, 0.36, 0.89, 2.04, and 4.54, respectively (Table 3).

The prevalence of asymptomatic carotid atherosclerosis is 22%.

## 4. Discussion

The most important finding of our study was that oscillometrically, the Arteriograph measured PWVao proved to be an independent marker of ACA. It is important to emphasize that age did not turn to be an independent factor of asymptomatic carotid atherosclerosis in this population. Although subjects with ACA were older compared to those without ACA, the difference (3.3 years) was modest. More importantly, the prevalence of ACA was 22% which is surprisingly high in this relatively young and apparently healthy population.

However, the other authors also found similar ACA prevalence in almost identical population. Roman et al. [20] investigated the relationship between asymptomatic carotid atherosclerosis and left ventricular hypertrophy in a large cohort of untreated hypertensive and normotensive patients free from overt cardiovascular or cerebrovascular disease. Sixteen percent of the 277 normotensive patients had ACA based on the carotid ultrasound examination. The mean age of the population was similar to ours (49 ± 12 years). In their study, age, SBP, and PP proved to be significantly higher in normotensive patients with asymptomatic carotid atherosclerosis.

In our study, the age independency of PWVao to reveal ACA maybe of paramount importance since most of cardiovascular risk scores are age-driven. Consequently, many older people are considered to have high CV risk despite of their normal cardiovascular status. However, subjects with low or moderate risk could be missed from the cardiovascular screening program because of their lower risk score driven by their younger age. Consequently, therapeutic intervention cannot be performed in time and fatal consequences may occur. However, several results confirmed that atherosclerotic process begins even at a very young age [4, 21]. Furthermore, it has to be emphasized that almost 60% of cardiovascular and cerebrovascular events occur among low-risk patients involving three-quarters of the population [22], where traditional risk factors do not identify the majority of patients who will develop cardiovascular disease in the next 10 years [23–25]. Consequently, measuring PWVao oscillometrically in an apparently healthy, middle-aged population to find individuals with asymptomatic atherosclerosis, who will take advantage from early intervention and aggressive treatment, may have great clinical importance.

We found 71% sensitivity for ACA by using the PWVao threshold value of 8.3 m/s based on the ROC curve in this middle-aged, apparently healthy, normotensive population, in which sensitivity seems to be acceptable. Our opinion could be supported by the fact that the sensitivity of the commonly used exercise ECG as a routine procedure to diagnose the presence of coronary artery disease in only 45–50% according to the 2013 ESC guidelines on the management of stable coronary artery disease [26]. Consequently, based on the fast, user-independent, easy application of the Arteriograph, this higher sensitivity seems to be acceptable for screening of asymptomatic atherosclerosis assuming that atherosclerosis is the systemic disease of the intimal layer of middle and large arteries potentially having long latent subclinical phase and can affect the carotid arteries as well as the coronary arteries [27]. The observed relatively low positive predictive value of 36% may result from the low prevalence of ACA positive cases in this study population. However, the high negative predictive value means that the majority of patients with PWVao lower than 8.3 m/s are unaffected from ACA. The relative risk and the odds ratio of the test proved to be 2.04 and 4.54, respectively, referring that the PWVao measurement seems to be a suitable screening tool for ACA.

The 8.3 m/s PWVao cutoff value for ACA in our study with middle-aged hypertensive patients was lower than it was recommended (10 m/s) by the ESC/ESH guidelines for the management of arterial hypertension as a marker of aortic stiffness and underlying arteriosclerosis [28, 29]. However, it must be emphasized that this higher cutoff value suggested by the guidelines does not refer only to ACA.

The relatively low age of our study population may point out that asymptomatic carotid atherosclerosis can be present even when PWVao is moderately elevated. Consequently, the 10 m/s general cutoff value may not be used for all the patients of different ages to estimate cardiovascular outcomes.

Our results were obtained by applying an ease-of-use, oscillometric method for measuring PWVao, which simplifies the PWVao measurement in the routine clinical work. This might be particularly useful for improvement of cardiovascular risk factor assessment in the primary care setting, where simplicity, user independency, and swiftness are mandatory requirements. It has to be emphasized that according to the 2016 European guidelines on cardiovascular disease prevention in clinical practice, the atherosclerotic plaque detection by carotid ultrasound as a risk modifier in CV risk assessment while IMT measurement is not recommended [26]. It is also worth mentioning that carotid ultrasound examination is hardly available in the primary care setting [7]. Despite the conclusion of the previously mentioned guideline [26], the SHAPE Task Force [2] has already encouraged the screening of atherosclerosis risk factors in younger vulnerable patients potentially having lower total cardiovascular risk based on traditional risk factors.

### 4.1. Limitations

Whether our results are merely specific to our studied, relatively small population, or may provide general conclusion?

Despite the lower patient number, we got the same results as other studies with more patients; consequently, this smaller sample seems to have the same effect on the surrogate end point. Moreover, our results are in line with other studies that proved a similar prevalence of asymptomatic atherosclerosis in the general population [30, 31]. Consequently, we can assume that using the 8.3 m/s cutoff value of PWVao to reveal ACA may be valid for the general population as well, but this presumption needs to be validated.

Another limitation of our study is the fact that it is cross-sectional, and no follow-up examination was performed. Nevertheless, asymptomatic carotid atherosclerosis, used as a surrogate end point in our study, has been already proven as a prognostic marker of both cardiovascular morbidity and mortality.

## 5. Conclusion

In conclusion, the study showed an independent association between PWVao, measured by a single upper arm cuff oscillometric method, and ACA in apparently healthy, middle-aged, normotensive population. The simplicity, user independency, and swiftness of the oscillometric method to measure PWVao may contribute to improve cardiovascular risk assessment even in relatively young, middle-aged, apparently healthy population. The solution may provide a future opportunity to implement the screening of this younger population for asymptomatic atherosclerosis potentially leading to the early identification of asymptomatic atherosclerosis and treatment to prevent the progression of the disease.

## Figures and Tables

**Table 1:** Descriptive data of the study population according to carotid artery atherosclerosis.

	*N*	All	Carotid atherosclerosis		*p*
			Negative	Positive	
*N*		236	185	51	
Age (years)	236	47 ± 8	47 ± 8	50 ± 8	0.009
Weight (kg)	236	76.0 ± 16.0	75.5 ± 15.7	77.9 ± 17.1	0.344
Height (cm)	236	172 ± 10	171 ± 9	173 ± 11	0.295
BMI (kg/m^2^)	236	25.7 ± 4.1	25.6 ± 4.1	25.8 ± 3.9	0.735
Serum total cholesterol (mmol/l)	134	5.6 ± 1.3	5.6 ± 1.3	5.9 ± 1.5	0.240
SBP (mmHg)	236	125 ± 10	125 ± 10	128 ± 9	0.048
DBP (mmHg)	236	75 ± 8	75 ± 8	76 ± 7	0.671
MAP (mmHg)	236	92 ± 7	92 ± 8	93 ± 7	0.228
PP (mmHg)	236	50 ± 8	50 ± 8	52 ± 8	0.049
HR (beats per min)	236	70 ± 9	70 ± 9	71 ± 10	0.380
PWVao (m/s)	236	8.2 ± 1.5	7.9 ± 1.3	9.3 ± 1.6	<0.001
Female/male	236	122/114	98/87	24/27	0.45
Smoker/nonsmoker	236	30/206	15/170	15/36	<0.001
			8.3%	29%	

BMI, body mass index; SBP, systolic blood pressure; DBP, diastolic blood pressure; MAP, mean arterial pressure; PP, pulse pressure; HR, heart rate; PWVao, aortic pulse wave velocity. Values are presented as mean ± standard deviation.

**Table 2:** Independent markers of asymptomatic carotid artery atherosclerosis by stepwise logistic regression analysis.

	Odds ratio and 95% confidence limits for 1 unit change	*p*
PWVao (m/s)	1.88 [1.44; 2.50]	<0.001
Smoker (yes/no)	3.79 [1.56; 9.22]	0.003
SBP (mmHg)	1.05 [1.001; 1.10]	0.046
DBP (mmHg)	0.94 [0.89; 0.99]	0.038

A stepwise logistic regression including age, gender, body mass index, smoking habits, systolic and diastolic blood pressure, heart rate, and PWVao showed aortic PWV, smoking, and systolic and diastolic blood pressure to be significant independent markers. PWVao, aortic pulse wave velocity; SBP, systolic blood pressure; DBP, diastolic blood pressure.

**Table 3:** Sensitivity and specificity of using 8.3 m/s as a cutoff value of aortic pulse wave velocity to reveal asymptomatic carotid atherosclerosis.

	Value	95% CI
Sensitivity	0.71	0.56–0.83
Specificity	0.65	0.58–0.72
Positive predictive value	0.36	0.27–0.46
Negative predictive value	0.89	0.82–0.94
Relative risk	2.04	1.56–2.66
Odds ratio	4.54	2.31–8.91

CI indicates confidence interval.

## Data Availability

The database we have built to prepare this manuscript was created based on the Hungarian/European Union law which was legally binding before 2018. The database contains the measurement per persons with name, date of birth, sex, and measurement values. As currently last year (2018) the GDPR (General Data Protection Regulation, https://ec.europa.eu/commission/priorities/justice-and-fundamental-rights/dataprotection/2018-reform-eu-data-protection-rules_en) was introduced in Hungary and in the European Union, we should have from each person who was involved in this study a written contest which complies to the GDPR to share publicly. We are currently working on it, but we can provide it to be available only after we will get from each person the written contest.
